# The *DTNBP1* (dysbindin-1) gene variant rs2619522 is associated with variation of hippocampal and prefrontal grey matter volumes in humans

**DOI:** 10.1007/s00406-012-0320-0

**Published:** 2012-05-13

**Authors:** S. Trost, B. Platz, J. Usher, H. Scherk, T. Wobrock, S. Ekawardhani, J. Meyer, W. Reith, P. Falkai, O. Gruber

**Affiliations:** 1 Department of Psychiatry and Psychotherapy, Centre for Translational Research in Systems Neuroscience and Clinical Psychiatry, Georg August University, Goettingen, Germany; 2 Department of Psychiatry and Psychotherapy, Ameos Clinic Osnabrueck, Osnabrueck, Germany; 3 Centre for Mental Health, County Hospitals Darmstadt-Dieburg, Groß-Umstadt, Germany; 4 Department of Neurobehavioral Genetics, University of Trier, Trier, Germany; 5 Department of Neuroradiology, Saarland University, Homburg, Germany

**Keywords:** Magnetic resonance imaging, Psychosis, Genetic susceptibility, Genetics, Hippocampus, rs1018381

## Abstract

*DTNBP1* is one of the most established susceptibility genes for schizophrenia, and hippocampal volume reduction is one of the major neuropathological findings in this severe disorder. Consistent with these findings, the encoded protein dysbindin-1 has been shown to be diminished in glutamatergic hippocampal neurons in schizophrenic patients. The aim of this study was to investigate the effects of two single nucleotide polymorphisms of *DTNBP1* on grey matter volumes in human subjects using voxel-based morphometry. Seventy-two subjects were included and genotyped with respect to two single nucleotide polymorphisms of *DTNBP1* (rs2619522 and rs1018381). All participants underwent structural magnetic resonance imaging (MRI). MRI data were preprocessed and statistically analysed using standard procedures as implemented in SPM5 (Statistical Parametric Mapping), in particular the voxel-based morphometry (VBM) toolbox. We found significant effects of the *DTNBP1* SNP rs2619522 bilaterally in the hippocampus as well as in the anterior middle frontal gyrus and the intraparietal cortex. Carriers of the G allele showed significantly higher grey matter volumes in these brain regions than T/T homozygotes. Compatible with previous findings on a role of dysbindin in hippocampal functions as well as in major psychoses, the present study provides first direct in vivo evidence that the *DTNBP1* SNP rs2619522 is associated with variation of grey matter volumes bilaterally in the hippocampus.

## Introduction

Schizophrenia is a common and severely disabling psychiatric disorder where heritability is of considerable relevance in the aetiology of the disease [9, 10, 43, 53]. One of the most replicated susceptibility genes for schizophrenia is the dysbindin-1 gene (*DTNBP1*; OMIM *607145) on chromosome 6p22.3. Straub and collaborators first reported an association of *DTNBP1* (dystrobrevin-binding protein 1, or dysbindin) with schizophrenia in a family-based association analysis of High-Density Schizophrenia Families in Ireland ([32], Irish Study of High-Density Schizophrenia Families ISHDSF) in 2002. Since then (and even before), numerous investigations have been realized to verify the role of *DTNBP1* as a schizophrenia susceptibility gene. While many genetic studies could replicate the association of *DTNBP1* with schizophrenia [17, 34, 55, 61, 71, 74, 75, 83, 87], others failed to confirm the finding of Straub and colleagues [50, 51, 67]. A meta-analysis authored by Li & He in 2007 only found a weak association of *DTNBP1* with schizophrenia [36].

On the other hand, there is emerging evidence that *DTNBP1* might also play a role in the genetics of affective psychosis. Large genetic case–control association studies of European [6, 19], Korean [31, 56], and multiplex Ashkenazi Jewish family samples [14] have shown a linkage of *DTNBP1* with bipolar disorder. However, in parallel to the state of research on schizophrenia, there are also negative association studies [58].

So far, more than 20 different SNPs (single nucleotide polymorphisms) have been reported to be significantly associated with schizophrenia and/or bipolar disorder, either individually or within a haplotype [19, 24]. However, there is widespread inconsistency among the associated *DTNPB1* haplotypes with a variety of combinations of SNPs and risk alleles reported throughout the literature [82], complicated even more by large genetic differences between major geographic populations [19, 24]. Thus, the question about the veritably underlying *DTNBP1* schizophrenia or other psychiatric susceptibility variants still remains [82].

*DTNBP1* encodes dysbindin-1 (β-dystrobrevin-binding protein or DTNBP1) [4, 64], a 40- to 50-kDa protein expressed neuronally [4] in many regions of the human brain [66]. DTNBP1 binds both α- and β-dystrobrevin, which are components of the dystrophin glycoprotein complex [4]. This complex is especially concentrated at postsynaptic densities [4] in diverse brain areas [4, 37], but only the β-dystrobrevin isoform is expressed in neurons [4]. By means of binding β-dystrobrevin, DTNBP1 interacts with the dystrophin glycoprotein complex at postsynaptic sites and might thereby alter neuronal synaptic mechanisms. However, independent of β-dystrobrevin and the dystrophin glycoprotein complex, DTNBP1 has also been found to be presynaptically located in glutamatergic neurons in the hippocampus [54, 69]. In this context, dysbindin-1 might play a role in glutamate signal transduction in the hippocampus formation [54].

*DTNBP1* is ubiquitously expressed throughout the brain [70, 80] and corresponding to its primary localization to neurons, *DTNBP1* mRNA has predominantly been detected in grey matter areas [80]. In the healthy adult brain, *DTNBP1* mRNA is most prominently expressed in the frontal cortex (especially in the dorsolateral prefrontal cortex), temporal cortex, hippocampus, caudate, putamen, nucleus accumbens, amygdala, thalamus, and midbrain [80]. Post-mortem brain analyses of patients with schizophrenia showed significantly reduced *DTNBP1* mRNA and protein expression in the dorsolateral prefrontal cortex (DLPFC) and midbrain [65, 66, 70] and in the hippocampal formation compared to healthy controls [54, 79]. Weickert et al. [79] reported a decrease in *DTNBP1* mRNA levels in schizophrenic patients particularly in the dentate gyrus and CA3 (cornu ammonis 3) of the hippocampus. This decline of mRNA was positively correlated with the expression of other synaptic markers known to be reduced in schizophrenia [79].

Alterations in dopamine and glutamate signal transduction are considered to be the neurochemical basis of the pathophysiology of schizophrenia [3, 12, 20, 47, 57, 73], but also play an important role in other psychiatric conditions [16, 24, 39]. In this context, genetic variation in *DTNBP1* might confer the risk of schizophrenia or other psychiatric disorders by mediating effects on synaptic structure and function of glutamatergic neurons, particularly in the hippocampus [48, 66].

Reductions in whole temporal lobe volumes and especially of the medial temporal lobe including the amygdala-hippocampus-complex are a major neuropathological finding in schizophrenia [62, 68, 76]. Most of all, reduced bilateral hippocampal volumes have been reported for chronic schizophrenia patients, but also for first-episode patients with psychosis and for patients with bipolar affective disorder [59, 68, 76]. Another principal neuropathological finding in schizophrenia is a reduced volume of prefrontal cortical regions [7, 22], which has also been described in bipolar patients to a lesser extent [49, 59]. Against this background, the aims of the present study were to directly investigate the effects of at-risk single nucleotide polymorphisms of the schizophrenia/psychiatric susceptibility gene *DTNBP1* on regional brain volumes in human subjects using structural MRI. We chose the two single nucleotide polymorphisms rs2619522 and rs1018381, which have been investigated in several genetic studies [14, 17, 19, 38], and even referred to as a set for tagging a high-risk haplotype, reported by van den Oord et al. [75] in the sample of the Irish study on high-density schizophrenia families. We hypothesized that there might be alterations in grey matter volume of the prefrontal cortex and hippocampal regions associated with the at-risk *DTNBP1* alleles.

## Materials and methods

### Subjects

Seventy-one (rs2619255) and seventy-two (rs1018381) subjects of a multi-diagnosis sample including nine healthy controls participated in the study (Table 1). In one case (male, 23 years, obsessive compulsive disorder), genotype analysis failed for SNP rs2619255. Apart from this, subjects were identical in both groups. Of the participating subjects, 22 were diagnosed with schizophrenia, 22 with bipolar affective disorder and 18 (rs2619255), 19 (rs1018381) with obsessive–compulsive disorder according to ICD-10 and DSM-IV criteria. The healthy controls exhibited no past or present psychiatric, neurological or medical disorder and had no positive family history of psychiatric disorders. Exclusion criteria in general were dementia, neurological illness, brain traumas, brain tumours and substance abuse. The patients were recruited from the Department of Psychiatry and Psychotherapy of the Saarland University Hospital between December 2003 and October 2006, and healthy controls were recruited from the hospital staff of the same institution and the local population. Written informed consent was obtained from all subjects prior to their inclusion into the investigation. The study was performed in accordance with the ethical standards laid down in the 1964 Declaration of Helsinki and was approved by the local ethics committee.

**Table 1:** Demographic and clinical data of study subjects

	rs2619522		rs1018381	
All subjects (*N*)	71		72	
Group comparison	GG/GT	TT	TT/CT	CC
Group size (*N*)	23 (4 GG; 19 GT)	48	10 (0 TT; 10 CT)	62
Gender (*N*)	10 M, 13 F	26 M, 22 F	4 M, 5 F	31 M, 31 F
Age (years ± SD)	35.3 (±11.1)	36.5 (±11.5)	34.2 (±10.4)	36.3 (±11.5)
Diagnosis: schizophrenia (*N*)	8	14	4	18
Diagnosis: bipolar disorder (*N*)	7	15	2	20
Diagnosis: OCD (*N*)	4	14	1	18
Control subjects (*N*)	4	5	3	6

*F* female, *M* male, *N* number, *SD* standard deviation

The mean age of all participants was 36 ± 11.3 years (rs2619255) and 35.9 ± 11.4 years (rs1018381). Age was ranging from 19 to 65 years. Thirty-five subjects were female, and all subjects were Caucasian (Table 1). The groups were not matched for diagnosis, age, and gender; therefore, these parameters were included into all voxel-based morphometric analysis as covariates of no interest. All participating subjects were scanned in the Saarland University Hospital.

### MRI data aquisition

Structural magnetic resonance imaging was performed on a 1.5-T scanner (Siemens, Erlangen, Germany). All participants underwent structural magnetic resonance imaging on the same scanner. A T1-weighted, MPRAGE/echo gradient sequence (TE = 4.42 ms, TR = 1,900 ms, TI = 700 ms, flip angle = 15°, FOV 256 × 256 mm) of 176 consecutive slices was acquired with a voxel size of 1 × 1 × 1 mm^3^. The magnetic resonance images were realigned in parallel to the anterior commisure—posterior commissure plane.

### Voxel-based morphometry and statistical analysis

Images were converted to DICOM format and processed using MATLAB and SPM5 software (Wellcome Department of Imaging Neuroscience Group, London, UK; http://www.fil.ion.ucl.ac.uk/spm). Images were corrected for bias and tissue classified. After segmentation into grey and white matter tissue classes, they were spatially normalized by using the standard SPM5 unified segmentation [2]. The normalized grey matter maps were modulated and finally smoothed with a 12-mm FWHM Gaussian kernel. All preprocessing steps were conducted using standard procedures as implemented in SPM5, in particular the voxel-based morphometry (VBM) toolbox.

Two-sample *t* tests were performed contrasting different groups according to their genotype. For both SNPs, we compared risk allele-carriers with homozygous non-risk allele-carriers. Confounding variables such as age, gender, and diagnosis were included in all VBM analysis as covariates of no interest to control for variability in these variables. We excluded all voxels with grey matter values of less than 0.05 (absolute threshold masking). One-tailed *t* contrasts were then generated with *p* < 0.001 (uncorrected, voxel-level) and extent threshold = 100 voxels.

### Genetic analyses

#### Single nucleotide polymorphism rs2619522 intron (G/T)

DNA was isolated from EDTA-blood samples and extracted according to the salting out procedure [46]. The following primers were used in the polymerase chain reaction (PCR): 5′-TGGGCCAGTGAAGTGAAAAT-3′ (forward) and 5′-TTGCAGCAAAACAGTACTCTCC-3′ (reverse).

The PCR was done in a 50-μl reaction mixture containing 100 ng of genomic DNA, 200 μM of each dNTPs, 10 pmol of each primer, 0.5 units Taq-polymerase, buffer D (KCl 50 mM, Tris–HCl 10 mM, Tween 20 0.025 %, BSA 0.025 mg/ml, and 2.0 mM MgCl_2_). The PCR had an initial temperature of 94 °C (5 min), followed by 35 cycles of denaturation (94 °C, 30 s), annealing (55.1 °C, 30 s), and extension (72 °C, 30 s). An extension period of 7 min at 72 °C followed the final cycle. The PCRs were done using an ABI GeneAmp^®^9700 cycler.

PCR products were digested at 55 °C using BseGI (FERMENTAS), separated on 2.0 % agarose gels (ROTI^®^GAROSE NEEO, Roth, Karlsruhe) and genotyped according to the resulting fragment lengths.

#### Single nucleotide polymorphism rs1018381 intron (C/T)

DNA was isolated from EDTA-blood samples and extracted according to the salting out procedure [46]. The following primers were used in the polymerase chain reaction (PCR): 5′-TGATTGAGGCTTTGGCTTTT-3′ (forward) and 5′-CCATGAGCATACCACAGCAC-3′ (reverse).

The PCR was done in a 50-μl reaction mixture containing 100 ng of genomic DNA, 200 μM of each dNTPs, 10 pmol of each primer, 0.5 units Taq-polymerase, buffer D (KCl 50 mM, Tris–HCl 10 mM, Tween 20 0.025 %, BSA 0.025 mg/ml, and 2.0 mM MgCl_2_). The PCR had an initial temperature of 94 °C (5 min), followed by 35 cycles of denaturation (94 °C, 30 s), annealing (55.1 °C, 30 s), and extension (72 °C, 30 s). An extension period of 7 min at 72 °C followed the final cycle. The PCRs were done using an ABI GeneAmp^®^9700 cycler.

PCR products were digested at 37 °C using NlaIII (NEB, Ipswich, MA, USA), separated on 2.0 % agarose gels (ROTI^®^GAROSE NEEO, Roth, Karlsruhe) and genotyped according to the resulting fragment lengths.

All genotypes were called by two individuals being blind to the clinical data.

### Genotype group classification

Participants were divided into two groups according to their genotype for both at-risk SNPs (rs2619522, rs1018381). For rs2619522 (*N* = 71), the G (guanine) allele has been defined as the minor allele and has been associated with schizophrenia, schizoaffective disorder [17] and bipolar disorder [6, 19]. For statistical reasons, we contrasted homozygous non-risk allele-carriers T/T (T = thymine; *N* = 48) with homozygous and heterozygous risk allele-carriers (*N* = 23; Table 1).

Regarding rs1018381 (*N* = 72), the T allele is referred to as the minor allele and has been associated with schizophrenia and schizoaffective disorder [17]. In the present study, we contrasted risk allele-carriers (C/T; C = cytosine; *N* = 10) with homozygous non-risk allele-carriers (C/C; *N* = 62). No subject was homozygous for the risk allele (T/T) (Table 1).

The *DTNBP1* allele distribution did not deviate from Hardy–Weinberg equilibrium for neither SNP (rs2619522: *p* = 0.2561; rs1018381: *p* = 1) [81].

## Results

### Single nucleotide polymorphism rs2619522

**Fig. 1:**
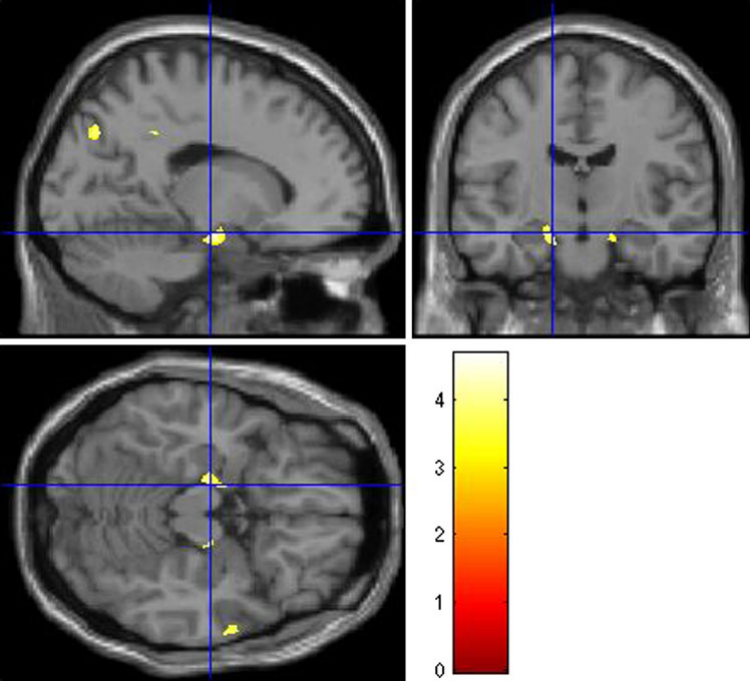
Effects of *DTNBP1* SNP rs2619522 on hippocampal grey matter volumes. Carriers of the G allele (risk allele) show significantly higher grey matter volumes in these brain regions than T/T homozygotes. *p* < 0.001 (uncorrected); extent threshold = 100 voxels

We found significant effects of the *DTNBP1* SNP rs2619522 on grey matter brain volumes. The major finding was in the left hippocampus with significantly altered grey matter volume associated with the risk allele (G allele; Fig. 1). This result remained significant after false discovery rate (FDR)-correction (*p*_FDR-corr_ = 0.042) and only scarcely missed the significance level for family wise error (FWE)-correction (*p*_FWE-corr_ = 0.069).

**Fig. 2:**
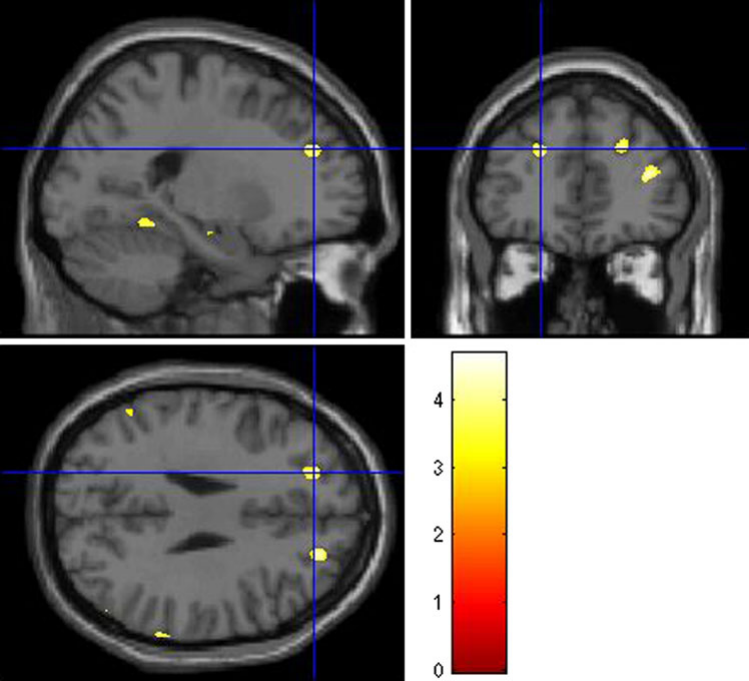
Effects of *DTNBP1* SNP rs2619522 on grey matter volumes of anterior prefrontal cortex. Carriers of the G allele (risk allele) exhibit significantly higher grey matter volumes in these brain areas than T/T homozygotes. *p* < 0.001 (uncorrected); extent threshold = 100 voxels

**Fig. 3:**
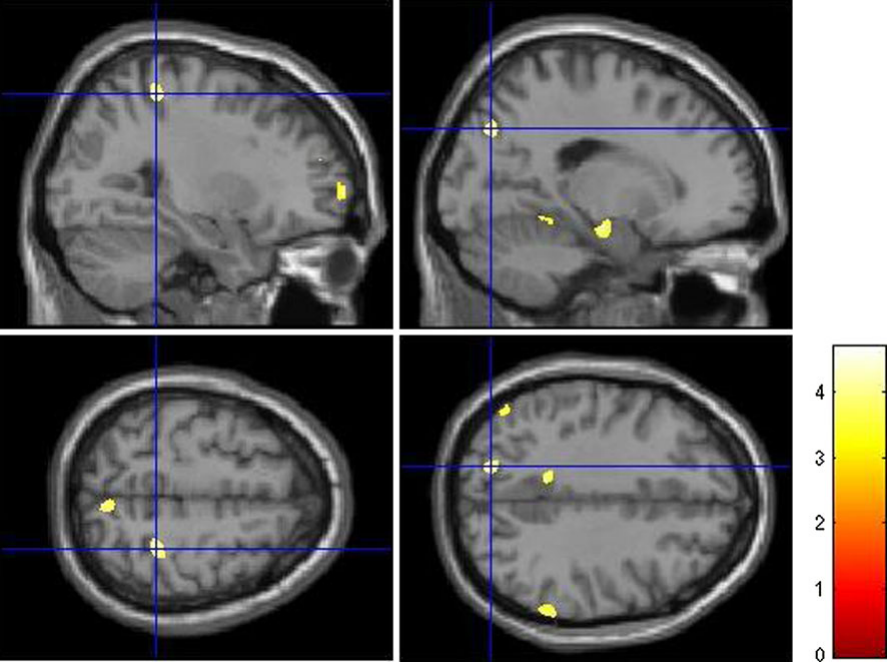
Effects of *DTNBP1* SNP rs2619522 on grey matter volumes of parietal cortices. G allele-carriers (risk allele) exhibit significantly higher grey matter volumes in these cortical regions than T/T homozygotes. *p* < 0.001 (uncorrected); extent threshold = 100 voxels

In addition, we found significant effects on a FDR-corrected level contralateral in the right hippocampus (Fig. 1) as well as in anterior prefrontal (Fig. 2) and intraparietal cortices (Fig. 3), in cortical regions of the temporal lobe and bilaterally in the cerebellum.

Carriers of the G allele (risk allele) showed significantly higher grey matter volumes in all these brain regions than T/T homozygotes (Table 2).

**Table 2:** *DTNBP1* SNP rs2619522 G/G + G/T > T/T

Anatomical regions	MNI coordinates	Statistical significance (*T* value)
Left hippocampus	−12/−13/−18	5.25
Right hippocampus	15/−16/−19	4.29
Left lingual gyrus	−20/−48/−11	4.17
Left lingual gyrus	−10/−48/−4	3.44
Left superior frontal gyrus	−16/53/32	3.83
Right superior frontal gyrus	10/59/25	3.86
Left middle frontal gyrus	−29/23/60	4.65
	−22/42/28	4.29
	−32/47/31	3.99
	−31/55/9	3.98
	−44/47/5	3.95
Right middle frontal gyrus	22/45/29	4.52
	39/42/16	4.36
	31/61/4	4.20
	43/48/0	3.81
	28/60/16	3.52
Right frontopolar gyrus	9/70/−6	3.93
Left inferior frontal gyrus	−57/20/9	4.82
Left inferior frontal gyrus, pars opercularis	−43/13/3	3.98
Right inferior frontal gyrus	41/23/5	4.36
	51/30/−11	4.05
Left postcentral gyrus	−57/−21/17	3.78
Right postcentral gyrus	67/−20/14	3.64
Left pregenual cingulate cortex	−5/54/2	3.66
Left anterior cingulate cortex	−3/33/32	3.56
(Right) anterior cingulate cortex	0/45/25	4.07
Left posterior cingulate gyrus	−11/−45/38	4.00
Right posterior cingulate cortex	6/−41/51	5.13
	5/−55/31	3.68
Left superior temporal gyrus	−46/−8/2	4.01
Right superior temporal gyrus	57/−34/15	4.15
Right superior temporal gyrus	61/−44/8	3.97
	70/−27/6	3.60
Left middle temporal gyrus	−58/−66/6	3.70
Right middle temporal gyrus	68/−6/−12	4.20
	70/−36/−5	4.01
Left angular gyrus	−48/−69/38	4.21
	−54/−59/31	3.92
Right angular gyrus	60/−45/39	4.65
Right supramarginal gyrus	52/−45/25	3.72
Left precuneus	−2/−56/50	3.56
Right precuneus	6/−69/56	4.29
Left superior occipital gyrus	−17/−77/37	4.99
Right middle occipital gyrus	59/−70/4	4.54
Right postcentral gyrus	55/−22/48	5.16
	29/−41/57	4.31
Right precentral gyrus	10/−7/75	4.25
	51/−9/35	3.83
	26/−31/67	3.50
Left cerebellum	−27/−38/−52	4.12
Right cerebellum	16/−57/−10	3.71
	23/−92/−29	3.70
Left inferior occipital gyrus	−13/−105/−3	3.92

*p* < 0.001 (uncorrected, voxel-level); extent threshold = 100 voxels

### Single nucleotide polymorphism rs1018381

There were significant alterations of grey matter volume in association with the risk allele T (genotype C/T) on a FDR-correction level. T-carriers exhibited significantly higher brain volume bilaterally in frontal cortices as well as in the left lingual gyrus, the right thalamus/pulvinar and the left cerebellum (Table 3).

**Table 3:** *DTNBP1* SNP rs1018381 C/T > C/C

Anatomical regions	MNI coordinates	Statistical significance (*T* value)
Right inferior frontal gyrus	49/14/22	4.08
Left lingual gyrus	−20/−50/−8	4.06
Right thalamus/pulvinar	8/−26/6	3.85
Left middle frontal gyrus	−32/52/7	3.80
Right middle frontal gyrus	35/46/14	3.75
Left cerebellum	−29/−54/−63	3.60

*p* < 0.001 (uncorrected, voxel-level); extent threshold = 100 voxels

## Discussion

In the present study, we found variations of grey matter brain volumes independently associated with two different single nucleotide polymorphisms of the *DTNBP1* gene, both together characterized as a set for tagging a schizophrenia high-risk haplotype reported by van den Oord et al. [75].

Regarding the SNP rs2619522, we found significantly higher grey matter volumes in several brain regions associated with the risk allele (G allele) [6, 17, 19]. The major finding was in the left hippocampus, which was significant on a FDR-correction level. Contralaterally, the right hippocampus also was significantly enlarged, although on a slightly lower significance level. Besides this main finding, we observed significant volume increases associated with the G allele in frontal and parietal cortices as well as in lateral and medial temporal areas.

Concerning the SNP rs1018381, we also found significantly higher grey matter volumes in association with the reported risk allele (T allele) [17]. Risk allele-carriers exhibited increased brain volumes bilaterally in frontal cortices, in the basal right thalamus/pulvinar and the left lingual gyrus.

Our results therefore complement the current state of research on schizophrenia and affective disorders with manifold findings relating to hippocampal and prefrontal morphology and function in the course of these psychiatric diseases [60, 62]. Schizophrenia has been associated with reduced hippocampal volume in patients as well as in their biologic relatives [5, 21, 28]. In bipolar disorder, findings of either enlarged [30] or reduced hippocampal volumes [13] have been described [60]. In addition, there are also several reports of asymmetric hippocampal volume alterations with emphasis on the left hippocampus in schizophrenia and individuals at ultra-high risk for psychosis [45, 77, 85], corresponding to our findings in association with rs2619522 (Table 2).

Hippocampal function, but also prefrontal and parietal cortical networks underlying higher cognitive functions, has been shown to be altered in schizophrenic and bipolar patients as well as in their healthy family members in several recent fMRI studies [11, 18, 23, 25, 26]. Moreover, cognitive impairment and abnormal executive functioning in schizophrenia and bipolar disorder have been reported in association with *DTNBP1* risk genotypes [8, 15], and deviating brain activation in prefrontal, temporal and parietal areas during cognitive and emotional tasks has been described in healthy carriers of *DTNBP1* risk alleles [33, 41, 42, 72, 84].

With this in mind, our findings of increased grey matter volumes in association with the *DTNBP1* risk alleles of both at-risk SNPs in the above-mentioned brain regions seem to be counterintuitive at first sight, especially with respect to the reported structural brain volume reductions in schizophrenia.

However, the pathophysiology of functional psychosis is still far from being understood in detail. According to the empirically well-supported dopamine hypothesis in schizophrenia [27], productive psychotic states are associated with dopaminergic hyperactivity in subcortical regions [29, 35, 44] and positive symptoms as well as memory deficits have been linked to dysfunctional hippocampal hyperactivity [86]. Along with this, there is a body of evidence of dynamic structural brain alterations over the course of time in schizophrenia [63], which applies to the hippocampal formation in particular [45, 78].

Intriguingly, Nickl-Jockschat and co-workers recently reported reduced white matter fractional anisotropy in the right perihippocampal region in healthy T allele-carriers of *DTNBP1* SNP rs1018381 [52]. At the same time, they also found elevated fibre tract integrity in bilateral frontal lobes in proximity to the bilateral medial frontal cortices, corresponding to our findings of higher grey matter volumes in association with the T allele (rs1018381) in these cortical brain areas.

Moreover, increased grey and white matter volumes in association with a schizophrenia risk allele-carrier status have also been described for a *Neuregulin*-*1* at-risk SNP in childhood-onset schizophrenia [1]. It is worth remarking that these risk allele-associated volumetric alterations were found equally in schizophrenia patients and healthy controls at the beginning of that prospective investigation, but differed in terms of structural neurodevelopment in the longitudinal course between groups to the detriment of the schizophrenia risk allele-carriers [1].

In this sense, our findings of increased grey matter volumes in critical cerebral regions associated with the here-reported risk alleles of *DTNBP1* might represent an endophenotype of a compromised brain [29], particularly vulnerable for developing further pathophysiological processes leading to clinical psychosis in the context of additional environmental stress. However, in which way and to what extent *DTNBP1* risk genotypes exactly modulate brain morphology and function still is a topic of current research.

It is noteworthy though that the present study, despite the relatively small sample size and its diagnostic heterogeneity, could provide such distinct findings on grey matter volumes in association with the two at-risk SNPs of *DTNBP1*. Given the phenotypic and genetic overlap of schizophrenia with bipolar disorder [40], the present results support existing evidence that genetic variation of *DTNBP1* may not only be a risk factor for schizophrenia, but also play a role in a broader context of psychiatric disorders including affective psychoses. Nevertheless, these results are limited by the diversity of the participating subjects and the findings will have to be replicated in a more homogeneous sample and with a higher number of subjects.

To conclude, compatible with previous reports on a role of the dysbindin-1 gene in hippocampal functions as well as in major psychoses, the present study provides first direct in vivo evidence that two risk alleles of *DTNBP1* show linkage to altered grey matter volumes in the human brain. The risk alleles of rs2619522 and rs1018381 are significantly associated with variation of grey matter volumes in prefrontal areas and the latter (rs2619522) even with altered grey matter volumes in parietal cortices and bilaterally in the human hippocampus.
